# The Efficacy and Safety of Chemotherapy in Patients With Nonsmall Cell Lung Cancer and Interstitial Lung Disease: A PRISMA-Compliant Bayesian Meta-Analysis and Systematic Review

**DOI:** 10.1097/MD.0000000000001451

**Published:** 2015-09-11

**Authors:** Yu Jie Chen, Ling Xiao Chen, Mei Xiang Han, Tian Song Zhang, Zhi Rui Zhou, Dian Sheng Zhong

**Affiliations:** From the Department of Oncology, General Hospital of Tianjin Medical University, Tianjin, P.R. China (YJC, DSZ); Department of Orthopedics, Tianjin Medical University General Hospital, Heping District, P.R. China (LXC); Department of Respiration, General Hospital of Tianjin Medical University, Tianjin, P.R. China (MXH); Internal Medicine of Traditional Chinese Medicine Department, Jing’an District Central Hospital of Shanghai, Shanghai, P.R. China (TSZ); and Department of Radiation Oncology, Fudan University Shanghai Cancer Center, Shanghai, P.R. China (ZRZ).

## Abstract

Chemotherapy plays a critical and venturous role against the co-morbidity of nonsmall cell lung cancer and interstitial lung disease (NSCLC–ILD).

We performed a Bayesian meta-analysis and systematic review to evaluate the safety and efficacy of the chemotherapy in NSCLC–ILD patients.

EMBASE, PubMed, the Cochrane Central Register of Controlled Trials, and clinicaltrials.gov (up to January 2015).

We included all study designs except case reports, all studies with NSCLC–ILD patients and all the possible chemotherapy regimens.

Quality was assessed by a components approach. We derived summary estimates using Bayesian method through WinBUGS (version 1.4.3, MRC Biostatistics Unit, Cambridge, UK).

Seven studies involving 251 patients with NSCLC–ILD were included in the meta-analysis. The treatment response (complete remission, 0; [partial remission, 39.1%; 95% credible interval [CrI], 32.6–45.7]; [stable disease, 36%; 95% CrI, 29.6–42.2]; [PD, 15.4%; 95% CrI, 11.3–19.8]; [nonevaluable, 6.4%; 95% CrI, 2.7–10.1]; [overall response rate, 41.3%; 95% CrI, 35.3–47.4]; [disease control rate, 77.7%; 95% CrI, 72.2–82.7]) were comparable to that of patients with NSCLC alone; the survival outcomes (median overall survival, median progression-free survival, and 1-year survival rate) were slightly worse, especially the lower 1-year survival rate. Platinum-based doublets as first-line chemotherapy may be related to higher incidence of acute exacerbation-ILD in first line chemotherapy (AE, 8.47%; 95% CrI, 5.04–12.6).

The data selection bias and small patient number make the meta-analysis of treatment response and conclusions generated from these data inaccurate.

The present meta-analysis suggests that chemotherapy might be an effective therapy for patients with NSCLC–ILD, but it might be associated with higher incidence of acute exacerbation.

## Abstract 2

Supplemental Digital Content is available in the text

## INTRODUCTION

Interstitial lung disease (ILD) is closely related to high incidence of lung cancer.^1^ Whether ILD causes lung cancer or vice versa, or both proceed, respectively, but resemble in characteristics, remains controversial. Recently, researches has shown that lung cancer and ILD may share common pathogenetic mechanisms,^2,3^ indications are that 2% to 8% of lung cancer patients yield to ILD.^3^

Although, chemotherapy plays an irreplaceable role against nonsmall cell lung cancer (NSCLC), patients with both NSCLC and ILD (NSCLC–ILD) taking chemotherapy may face risks of chemotherapy-related acute exacerbation of ILD (AE-ILD).^4^ In a retrospective study from Japan, 9 out of 104 patients (8.7%) developed chemotherapy-related AE-ILD during the first-line platinum-based treatment^5^; in another prospective study, combining paclitaxel weekly treatments with carboplatin caused AE-ILD in 1 out of 18 patients (5.6%).^6^ Specifically, this clinical situation is fatal and of poor prognosis.^7,8^ The question arises as to whether chemotherapy regimens are efficacious and safe for the co-morbidity population.

So far, neither consensus has been reached nor have enough evidence been presented to support an optimal treatment strategy for NSCLC–ILD patients^9,10^—these patients are usually excluded by most clinical trials and the relevant studies are largely single-armed. Therefore, we aimed to perform a Bayesian meta-analysis and systematic review to evaluate the safety and efficacy of the chemotherapy in patients with NSCLC–ILD.

## METHODS

This article followed Preferred Reporting Items for Systematic Reviews and Meta-Analyses (PRISMA) guidelines.

### Eligibility Criteria

Studies were selected according to the following criteria:

1. Study designs: we included all study designs except case reports.
2. Participants: we included all studies with NSCLC–ILD patients.
3. Interventions and comparisons: we included all the possible chemotherapy regimens. If studies in which patients received radiotherapy before chemotherapy, we would exclude them because radiotherapy might cause radiation pneumonitis, or we would extract the data of patients without receiving radiotherapy.
4. Outcomes: complete remission (CR), partial remission (PR), stable disease (SD), progressive disease (PD), nonevaluable (NE), overall response rate (ORR), disease control rate (DCR), 1-year survival rate, median overall survival (mOS), median progression-free survival (mPFS), the incidence of AE-ILD related to first-line chemotherapy and toxicities.
5. Time and settings: no restrictions were set on the duration of follow-up and types of settings.

### Search Methods and Study Selection

We searched EMBASE (from 1974 to January 2015), PubMed (from 1966 to January 2015), the Cochrane Central Register of Controlled Trials (The Cochrane Library, most recent issue), and clinicaltrials.gov. Keywords and MeSH terms were related with chemotherapy, ILD and NSCLC. A PubMed search strategy was in Supplemental File 1, http://links.lww.com/MD/A407. We also reviewed every reference listed in the included studies, all related reviews and guidelines trying to avoid any previously ignored papers.

Two authors independently made the selection based on title and abstract. Any disagreement between 2 authors was resolved by discussion. If there was no consensus, a third reviewer (DSZ) was consulted.

### Data Collection and Quality Assessment

We extracted study, country, setting, sex, age, study design, and sample size. The efficacy outcomes were treatment response (CR, PR, SD, PD, NE, ORR, and DCR) and survival outcomes (1-year survival rate, mOS, and mPFS); the safety outcomes were the incidence of AE-ILD related to first-line chemotherapy and toxicities. For missing data, we would contact with the respective corresponding authors. Quality assessment was performed by a components approach proposed by Viswanathan et al.^11^ The approach included 7 domains and the judgments for all domains were yes (Y), no (N), and not reported (NR).

### Data Synthesis

Dichotomous variables were determined by using odds ratio (OR) with 95% credible interval (CrI). Bayesian methodology was used to perform meta-analyses through WinBUGS (version 1.4.3, MRC Biostatistics Unit, Cambridge, UK).^12^ The posterior parameters were calculated by Markov chain Monte Carlo methods. The convergence was assessed by visually checking trace and Brooks–Gelman–Rubin plots.^13^ Once convergence was determined, the initial burn-in iterations were discarded and a further 100,000 iterations were used to obtain our results. For mOS and median PFS which did not have confidence interval (CI), we used Shapiro–Wilk test through R (version 3.1.1, R Foundation for Statistical Computing, Vienna, Austria) to test whether the data were normally distributed. If the data were normal, mean and 95% CI would be determined to describe the outcomes, otherwise median and interquartile were used. For the data that could not be integrated, we described them in a narrative way. Subgroup analysis was performed based on chemotherapy regimens. Publication bias could be tested if 10 or more studies were included.^14^ Sensitivity analysis could be conducted if there were studies which had obvious heterogeneities. We performed hypothesis testing to compare the results of our analysis with ECOG 1594 trial and Scagliotti trial. Chi-squared test was used to compare 2 rates by R (version 3.1.1, R Foundation for Statistical Computing, Vienna, Austria). Z test was performed to compare 2 groups of data with their CI through Excel. For those data without CI, we estimated their CI in 2 ways: when the range of rate was from 0.3 to 0.7, we used approximate normal computation through Excel; otherwise, we used arcsine square root transformation based on Stata software (version 12.0, StataCorp, College Station, TX). The detailed computing process and theory were described before.^15^*P* < 0.05 was considered as significant statistical differences. Ethical approval was not necessary because our research collected data from literatures instead of patients.

## RESULTS

### Description of the Included Studies

**FIGURE 1:**
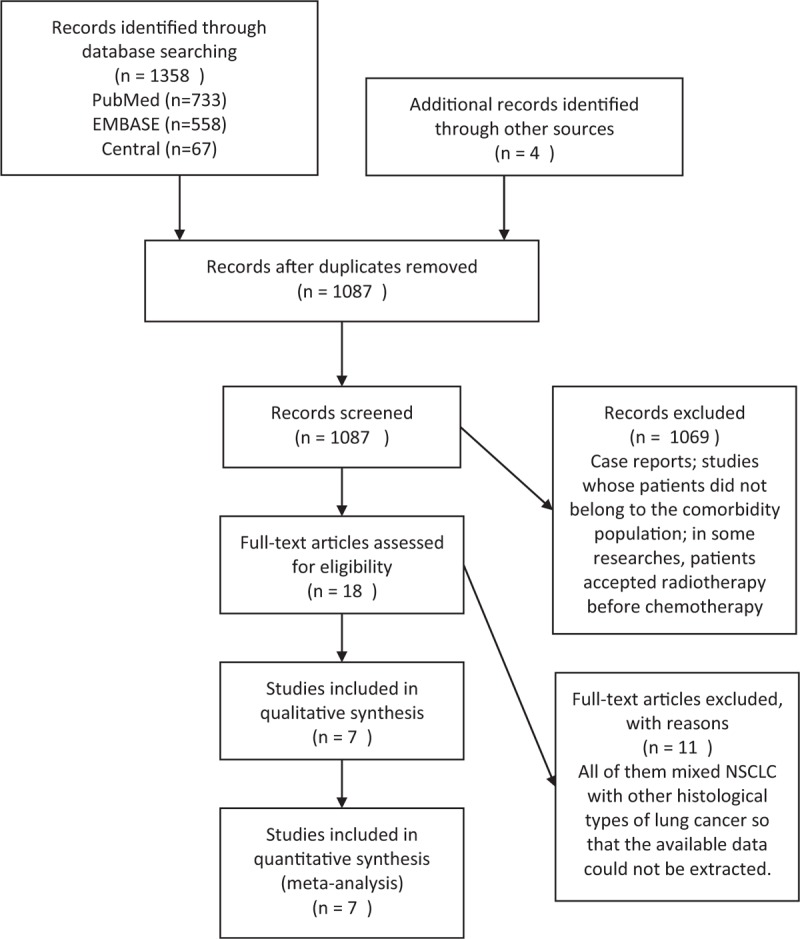
Search, inclusion, and exclusion flow diagram.

The process from identification to inclusion is shown in the PRISMA flow diagram (Figure 1). By January 2015, a total of 7 studies met the inclusion criteria, and the data were included in the qualitative synthesis and meta-analysis.^5,6,16–20^ Eleven studies focused on lung cancer were excluded because data of NSCLC–ILD patients could not be extracted.^4,21–30^

### Study Features

The characteristics of studies are shown in Table 1. Seven retrospective studies had been conducted in Japan and Korea between 2010 and 2015. The median patient age and sex ratio of each study were closely aligned. The majority of total 251 patients with NSCLC–ILD accepted platinum-based chemotherapy as first-line treatment without other interventions (eg, thoracic radiotherapy or molecular targeted drug) that may cause AE-ILD. Of these patients, most were with good performance status (PS 0–2), except one with PS 3. The patients’ clinical stages of NSCLC were mainly IIIA, IIIB, and IV or recurrence after surgical resection, the exception was 4 patients, 2 each with stages I and II. Adenocarcinoma accounted for approximately 53% in our patients. The histological types of ILD was omitted, since we were concerned more about the overall features of NSCLC–ILD population, besides, some details were not provided in records studied.

**TABLE 1:**
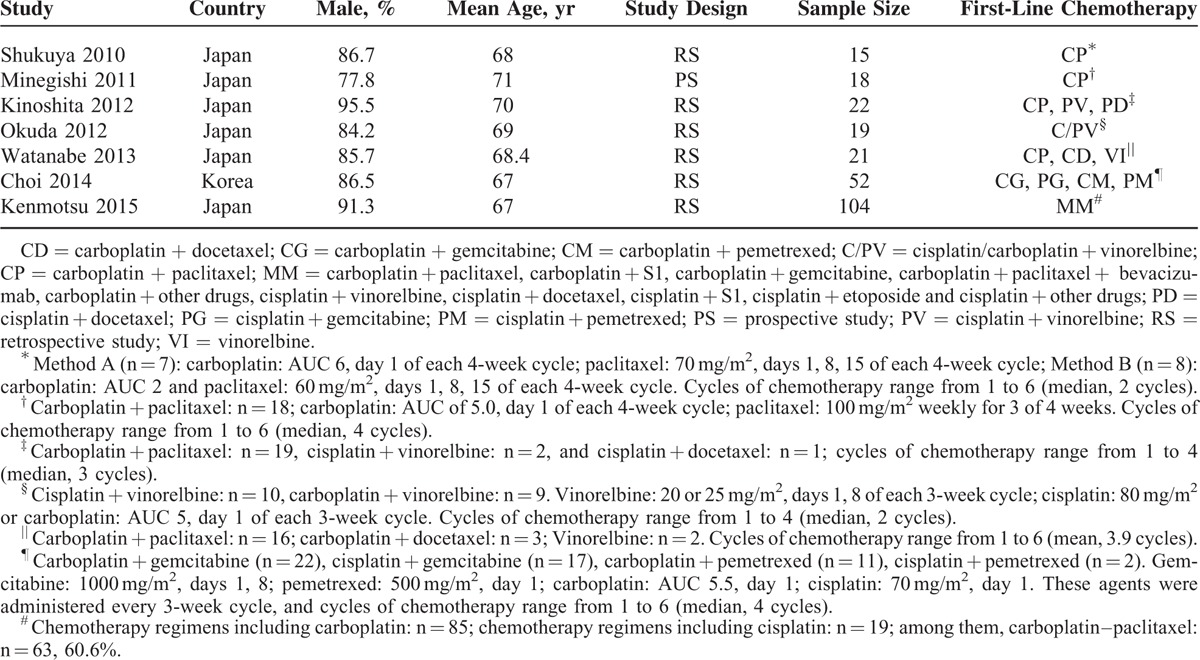
Characteristics of Included Studies

### Quality Assessment

Table 2 shows the result of our quality assessment. The outcomes measured in the included studies were predefined. All studies used a valid and reliable gambling measure with assurance that they did not accept any sponsorship from the gambling industry and that their observational studies underwent naturalistic treatment. There were no studies with appropriate comparison groups. Only 3 studies described consecutive or random recruitment of participants.^5,16,19^ Four studies ruled out any influence from a concurrent intervention or an unexpected exposure that might bias results.^5,16,17,20^

**TABLE 2:**
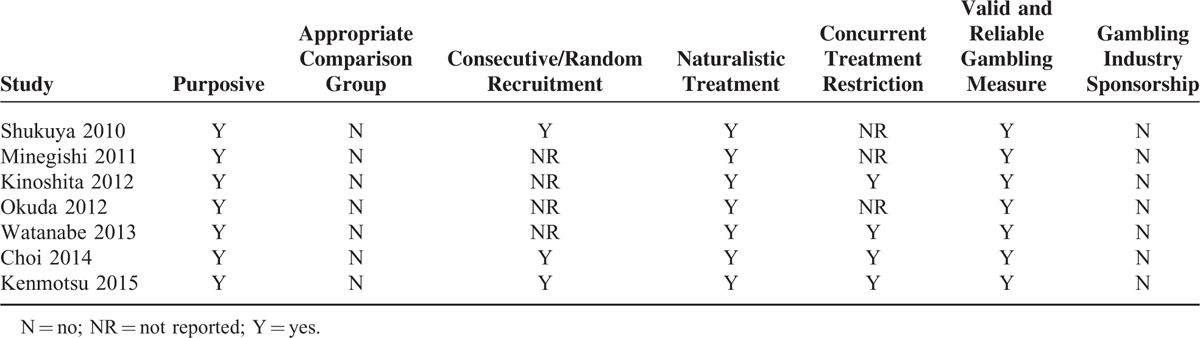
Risk Assessment of Included Studies

## EFFICACY OUTCOMES

### Treatment Response

A total of 5 studies including 212 patients reported CR and PR, and the pooled estimate was 0, and 39.1% (95% CrI, 32.6–45.7), respectively.^5,16–19^ Seven studies covering 251 patients evaluated SD, PD, and NE,^5,6,16–20^ the pooled estimates were 36% (95% CrI, 29.6–42.2), 15.4% (95% CrI, 11.3–19.8), and 6.4% (95% CrI, 2.7–10.1), respectively. The combined results of these 7 studies found ORR at 41.3% (95% CrI, 35.3–47.4), and DCR at 77.7% (95% CrI, 72.2–82.7).^5,6,16–20^

### Survival Outcomes

Five studies including 125 patients evaluated 1-year survival rate and the pooled estimate was 29.4% (95% CrI, 22.0–37.3).^6,16,18–20^ The pooled estimate of mOS and median PFS from 7 studies were OS at 8.5 months (95% CI, 6.5–10.5) and PFS at 4.4 months (95% CI, 3.3–5.5).^5,6,16–20^

## SAFETY OUTCOMES

### The Incidence of AE-ILD Related to First-Line Chemotherapy

Five studies involving 223 patients were included in the meta-analysis to evaluate the incidence of AE-ILD related to first-line chemotherapy and the result was 8.47% (95% CrI, 5.04–12.60).^5,6,16–18^ One study used “rapid deterioration” for acute respiratory worsening, which had been caused by various conditions rather than “AE-ILD” was excluded. The occurrence of rapid deterioration was found in 9 of 21 patients (42.9%) after chemotherapy and 8 of these 9 died.^20^

## TOXICITIES

Four studies reported the toxicities of palliative chemotherapy in NSCLC–ILD patients, stages IIIA–IV or recurrence after surgery.^6,18–20^ Of the toxicities (Table 3), the most common hematological grades 3 and 4 adverse effect was neutropenia—41.1% (30 of 73 patients); nonhematological adverse events (eg, renal dysfunction, peripheral neuropathy, nausea, fatigue, and so on) were mild in general, except pneumonities—9.6% (7 of 73 patients), hypoxia—6.8% (5 of 73 patients), and infection—5.5% (4 of 73 patients) were with grade 3 or higher.

**TABLE 3:**
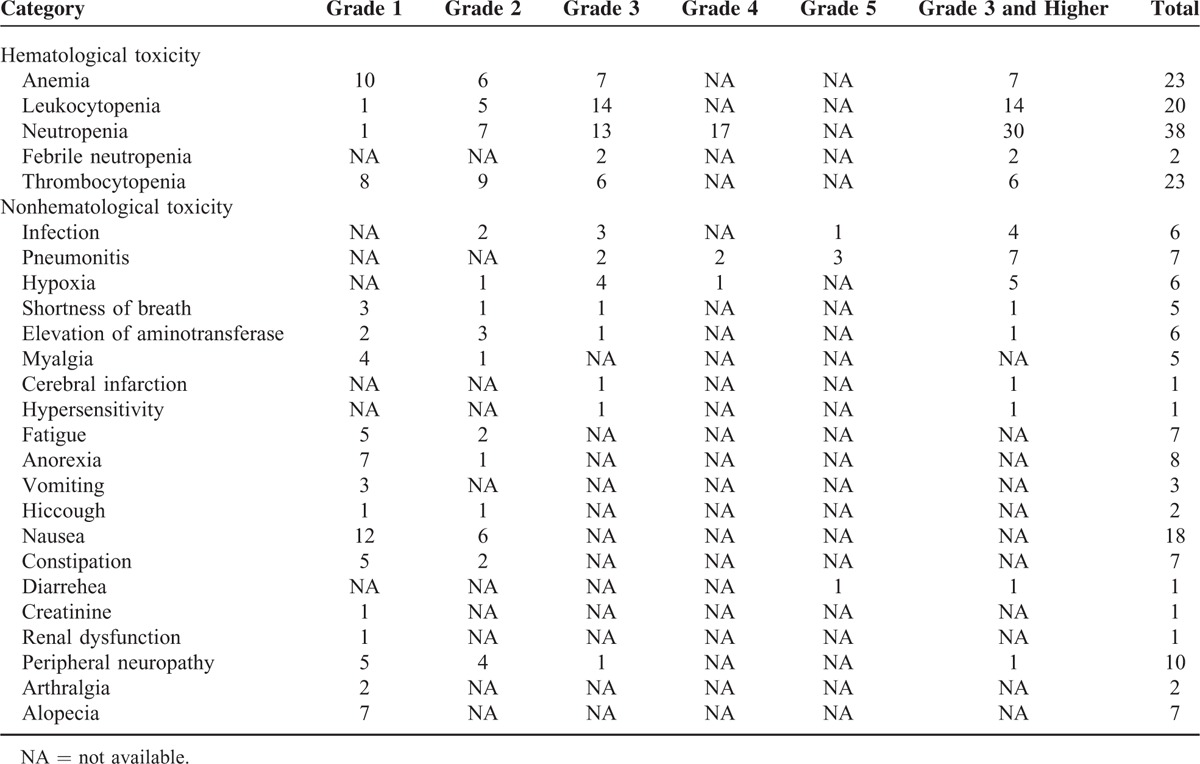
Toxicities Other Than Acute Exacerbation of Interstitial Lung Disease Related to First-Line Chemotherapy (n = 73)

### Sensitivity Analyses

One study included some patients who had accepted nonchemotherapy (12 of 104 patients), but we could not separate their information, except the incidence of AE-ILD related to first-line chemotherapy (it had been reasonably analyzed).^5^ We therefore performed a sensitivity analysis by excluding the study. The results were almost the same as with the study included.

### Subgroup Analyses

Only the data (treatment responses and the incidence of AE-ILD related to first-line chemotherapy) of carboplatin + paclitaxel regimen could be analyzed. The results were as follows:

- no patients with CR were observed.
- PR was achieved in 27 of 78 patients.
- 35 of 96 experienced SD.
- 13 of 96 were with PD.
- NE were found in 10 of 96.
- ORR was reported in 47 of 112.
- DCR in 87 of 112.

The pooled estimates of CR, PR, SD, PD, NE,ORR, and DCR were 0, 35.3% (95% CrI, 17.3–55.6), 32.8% (95% CrI, 13.6–49.9), 14% (95% CrI, 7.1–22.2), 12% (95% CrI, 3.6–23.7), 43.9% (95% CrI, 31.9–58.1), and 77.1% (95% CrI, 65.5–87.1), respectively.

The incidence of AE-ILD related to first-line chemotherapy was 9.5% (95% CrI, 3.7–16.6).

## DISCUSSION

### Summary of Main Results

This is the first meta-analysis and systematic review to evaluate the efficacy and safety of the first-line chemotherapy in patients with both NSCLC and ILD (NSCLC–ILD). The efficacy of treatment was proved by tumor response rate (eg, CR, PR, SD, PD, ORR, and DCR) and the survival outcomes (1-year survival rate, mOS, and mPFS). Indicators, such as the incidence of AE-ILD related to the first-line chemotherapy and toxicities, were used to demonstrate the adverse effects of chemotherapy.

### Consistency and Discrepancy in the Current Literature

The combination of platinum with a third-generation chemotherapy agent is considered as the standard first-line treatment for patients with NSCLC,^31^ and neither of 2 regimens differ in impact on survival.^32^ In the ECOG 1594 trial, the therapeutic potential of 4 platinum-based combine chemotherapy regimens (cisplatin and paclitaxel, cisplatin and gemcitabine, cisplatin and docetaxel, and carboplatin and paclitaxel) was elucidated: CR: <1%, PR: 19%, SD: 21%, PD: 45%, NE: 15%, ORR: 19%; mOS: 7.9 months (95% CI, 7.3–8.5), and median time to progression (mTTP): 3.6 months (95% CI, 3.3–3.9).^33^ We attempted to determine whether NSCLC–ILD patients was inferior to NSCLC patients without ILD, regarding the efficacy of first-line chemotherapy.

In our study, both tumor response and survival of the NSCLC–ILD patients seem to be superior to the patients with NSCLC alone in ECOG 1594 trial, except 1 year survival rate. However, given the different composition of histological types in NSCLC population, which were not mentioned in ECOG 1594 trial but might affect the prognosis. Thus, we conducted a hypothesis test between ECOG 1594 and the study of Scagliotti et al that had a similar constitution with our study (Table S1, http://links.lww.com/MD/A407), and significant statistical differences existed in the result. Therefore, we compared our data with study of Scagliotti et al. In the study, patients accepted gemcitabine and cisplatin (GC), paclitaxel and carboplatin (PCb), vinorelbine and cisplatin (VC), respectively.^34^ The total treatment response was evaluated as: CR: <0.5%, PR: 30% to 31%, SD: 31% to 40%, PD: 17% to 18%, NE: 13% to 23%, ORR: 30% to 32%. As with the time-to-event outcomes, the mOS of the GC, PCb, and VC groups were 9.8 months (95% CI, 8.6–11.2), 10.0 months (95% CI, 9.0–12.5), 9.5 months (95% CI, 8.3–11.0), respectively; for mPFS were 5.3 months (95% CI, 4.4–6.3), 5.5 months (95% CI, 4.6–6.4), 4.6 months (95% CI, 3.9–5.6), respectively; for 1-year survival rates were 37%, 43%, and 37%, respectively. According to the hypothesis test (Table S2, http://links.lww.com/MD/A407), our data of treatment response were largely consistent with the study performed by Scagliotti, and PR, ORR, and DCR were slightly favorable. mOS, mPFS, and 1-year survival rate were slightly worse, though the poor survival was not statistically significant, which may be result from insufficient power caused by the limited number of studies.

We considered that the poorer prognosis of patients in our study may be associated with the acute exacerbation (AE) during course of ILD instead of futile chemotherapy. We suspected this for 3 reasons. First, we minimized interferences such as: clinical stage, constituent ratio of sample, and the PS during comparison. Second, based on the lower 1-year survival rate of our patients, we infer that more patients died due to drug-related AE within the first year after accepting chemotherapy. Finally, the superior tumor responses manifested the effect of chemotherapy to depress tumor. These facts indicated that the poorer survival was related to the developing of AE related to first-line chemotherapy which shortened the life time other than the ineffective chemotherapy in NSCLC–ILD patients.

The preexisting ILD have been proved to be the risk factor for AE related to first-line chemotherapy.^26,35^ To estimate the safety of the first-line chemotherapy in NSCLC–ILD patients, the incidence of AE in this study was calculated. According to previous studies, idiopathic pulmonary fibrosis (IPF) patients likely to develop AE in all types of ILD during the nature course.^36,37^ Another study showed the incidence of acute exacerbation in IPF patients was 8.5% in 1 year period and 9.6% in 2 years after diagnosis.^38^ In our study, the incidence of AE-ILD, related to the whole treatment process, ranged from 13.5% to 25%,^5,6,16–18^ and was much higher than that during its nature course. Further, the incidence rate of AE-ILD only related to the first-line chemotherapy was 8.47% (95% CrI, 5.04–12.60), within <4 months,^6,16,18,19^ that was similar to the incidence of acute exacerbation of IPF within 1 year during its nature course. Furthermore, there was no evidence to prove that lung cancer was a risk factor of AE-ILD, so we supposed that the development of AE during the nature course was with equal incidence in patients with NSCLC–ILD and ILD alone. Based on above reasoning, we interpret that first-line chemotherapy may be associated with higher incidence of AE-ILD. To investigate which first-line chemotherapy regimen had related to the lowest incidence of AE-ILD, we performed a subgroup analysis, and the only available data of carboplatin and paclitaxel regimen fitted well with the data of the entire population. This demonstrated carboplatin and paclitaxel regimen would not be related to higher incidence of AE.

Toxicities are also considered as indicators to assess the safety of therapies in patients with NSCLC–ILD. Thus, we summarized the toxicities observed during treatment (Table 3). According to the previous study, the toxicities of platinum-based doublets regimen could not be ignored in NSCLC patients without ILD.^39,40^ Based on our result, the generating of hematological and nonhematological adverse effects (grade 3 or higher) was primarily due to the myelosuppression that could explain the developing of neutropenia and the consequent serious infection. Compared with the study of Hotta et al, the incidence of neutropenia in patients with NSCLC–ILD is similar to those without ILD.^40^ All toxicities above made the appearance of hypoxia reasonable.

## LIMITATIONS

First, although there were no restrictions by the language form, we only included papers written in English, which meant selection bias might exist. Due to the limited number of included studies, we could not perform the funnel plot to test the publication bias. Second, although we used the Bayesian method to narrow the 95% CrI, the data selection bias and small patient number definitely make the meta-analysis of treatment response and conclusions generated from these data inaccurate. Third, the lacking of appropriate control groups in our included studies made us hard to draw definite conclusions. In addition, we used summary data instead of individual patient data, which might lead to the loss of some covariates at the individual patient level. Moreover, 1 study included some patients who accept other treatment that might affect the results; therefore, we performed a sensitivity analysis by excluding the study and the results were stable. Finally, the external validity of the results might be limited due to 2 reasons: first, 6 studies were performed in Japan and 1 was in Korea. Second, considering mean age of included studies were larger than 65 and most of patients were male, our results should be applied to old males only.

## CONCLUSION

Chemotherapy might be an effective therapy for patients with NSCLC–ILD, but it might associated with higher incidence of AE. To ensure the efficacy and safety of chemotherapy, more clinical trials with control groups should be performed in a larger more widespread patient population. Further, to investigate the optimal chemotherapy regimen, head-to-head trials should be performed.
